# Constitutionality of Legislation Restricting Medical Practice

**Published:** 1914-06

**Authors:** 


### Constitutionality of Legislation Restricting Medical Practice

Certain recent legislation suggests that there has been a close approach to the limit of professional control if not an actual trespass on vested rights. Drug habits are a recognized evil but drugs susceptible of such abuse are equally well recognized as a necessary part of the armamentarium. Physicians are licensed by states. Has the national government the right to interfere with this license, either by imposing restrictions which consume time, entail trouble and render even a conscientious physician liable to criminal prosecution and punishment for technical violations; or by interference with the accepted doctrine of privileged communications; or, according to certain extreme interpretations of the law, by actually removing his license to practice certain accepted phases of his art?

Has even a state, after granting a license, the right to pass similar laws which are retroactive, not of course in their application to individual acts, but as regards the general privileges granted?

Now is the time for the medical profession to consider these matters seriously and broadly. Certain arbitrary restrictions are necessary in civilized life, and it is an accepted principle, that ignorance of the law is not an excuse for its violation. Yet, until recently, it was possible for any man of good intentions and common sense to follow his vocation and daily life without apprehension that he was liable to arrest for violation of a law,except in emergent conditions that naturally suggest inquiry as to legal requirements. This practical expression of individual liberty has been superseded already, for many citizens. In particular, the medical profession is hampered by so many legal details that it requires constant study and anxiety to avoid infringing their limitations or omitting their demands.

We have readily granted the necessity of reporting actively contagious disease; even of reporting tuberculosis and venereal disease in certain cases. We concede the danger of narcotic drugs, but is this danger greater than for arsenic, mercury, phosphorus and many others? The right of a surgeon to perform certain operations, without delays dangerous to life, has already been questioned by the introduction of ordinances and bills. Every restrictive law passed, establishes a precedent for others. Where is the process of restriction going to stop?

